# Stability and Optoelectronic Properties of Two-Dimensional Gallium Phosphide

**DOI:** 10.1021/acsomega.4c03861

**Published:** 2024-08-09

**Authors:** Elisangela da Silva Barboza, Kessia L. M. Cruz, Ramon S. Ferreira, Alexandre C. Dias, Erika N. Lima, Diego R. da Costa, Teldo A. S. Pereira

**Affiliations:** † Instituto de Física, Universidade Federal de Mato Grosso, 78060-900 Cuiabá, Mato Grosso, Brazil; ‡ Departamento de Física, Universidade Federal do Piauí, 64049-550 Teresina, Piauí, Brazil; § National Institute of Science and Technology on Materials Informatics, 13083-886 Campinas, Brazil; ∥ Institute of Physics and International Center of Physics, University of Brasília, Brasília 70919-970, Distrito Federal, Brazil; ⊥ Departamento de Física, Universidade Federal do Ceará, Campus do Pici, 60455-900 Fortaleza, Ceará, Brazil; # Department of Physics, University of Antwerp, Groenenborgerlaan 171, B-2020 Antwerp, Belgium

## Abstract

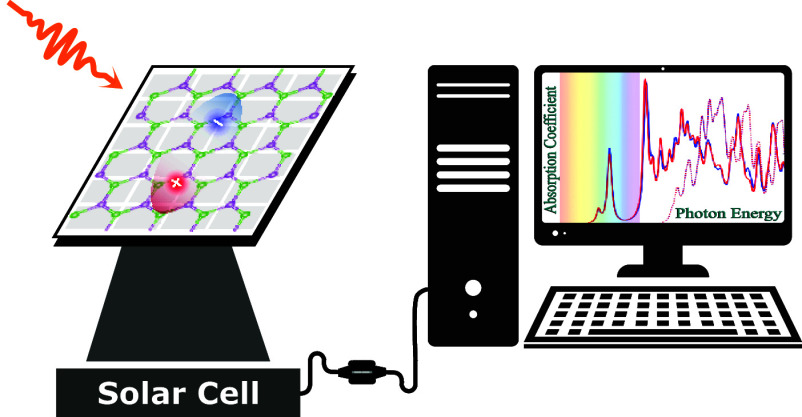

Using first-principles calculations, density functional theory, and the tight-binding method, we investigate the optoelectronic properties of two-dimensional gallium phosphide (2D GaP). Our investigation covers electronic properties, such as band structure and electronic band gap, and optical properties, including absorption spectra, refractive index, and reflectivity, considering excitonic effects. Additionally, structural aspects such as stability, elastic properties, and Raman and infrared spectra are also analyzed. This comprehensive study brings up valuable insights into 2D GaP physics, evincing the key features that make it a potential material for optoelectronic applications, such as photodetectors and solar cells.

## 1 Introduction

Conventional semiconductor materials have held an important position at the forefront of technological device applications for a long time, thanks to their tunable physical quantum properties.^1^ The list of new promising materials has increased over the years.^2−4^ A potential candidate of the semiconductor zoo is gallium phosphide (GaP) that has played an important role since the 60s in the photonics area, composing a basis for a series of light-emitting devices in the green frequency.^5−7^ Despite its large indirect band gap energy of 2.26 eV at room temperature and in the wurtzite phase,^8^ the GaP has a large refractive index, greater than 3, enabling strong optical confinement and leading to a pronounced nonlinear optical response, with high second χ^(2)^ and third χ^(3)^ order optical susceptibility.^9,10^

The advent of graphene’s^11^ mechanical exfoliation in the first decade of the 21st century prompted the advancement of crystal obtaining in two-dimensional (2D) format, boosting the search for new lamellar materials that, unlike graphene, exhibited a non-null energy gap. Despite the escalating interest in 2D materials and their potential applications, investigations into the electronic, optical, and structural properties of 2D GaP have been conspicuously limited.^12−16^ In view of the lack of substantial comprehension of the 2D GaP physical properties, this work aims to fill this knowledge gap by exploring the structural, electronic, and optical aspects of monolayer GaP.

To illustrate one of a few studies on 2D GaP, it is relevant to cite ref (16) that presents a theoretical ab initio-based investigation into the electronic properties of 2D hexagonal planar GaP in its bulk phase. The study is a short paper that succinctly delves into the band structure, the total density of states (DOS), and the projected density of states (PDOS). It also briefly discussed geometric insights as integral aspects of the electronic properties. In addition, by using density functional theory (DFT) calculations, ref (14) shows that the band gap of monolayer GaP can be tuned by applying external biaxial mechanical strain and perpendicular electrical field. They demonstrated that monolayer GaP has maximum values of optical conductivity in the ultraviolet energy range that prevails in their use as ultraviolet detectors. To our knowledge, unlike the current work here, no study reported in the literature has taken into account any excitonic effect in the optoelectronic properties, of monolayer GaP, as well as no general structural investigation has been conducted exploring deeply the lattice stability and its elastic properties, including Raman and infrared spectra, as will be discussed here.

The study is organized as follows: in Section 2, we briefly present the computational details employed within the first-principles frameworks assumed here. Results are discussed in Section 3 in view of structural (Section 3.1), electronic (Section 3.2), elastic (Section 3.3), vibrational (Section 3.4), excitonic (Section 3.5) aspects, as well as insights into solar efficiency are given in Section 3.6, aiming for its usage as photovoltaic devices.

## 2 Computational Details

Our calculations were performed using DFT, with the exchange-correlation (XC) functional proposed by Perdew–Burke–Ernzerhof (PBE)^17^ to optimize the crystal structure and compute the electronic properties, phonons, ab initio molecular dynamics, Raman and infrared spectra. The electronic band gap description was improved using the hybrid XC-functional proposed by Heyd–Scuseria–Ernzerhof (HSE06),^18,19^ by means of the self-interaction errors that result in a band gap underestimation at PBE framework.^20,21^ Based on refs (22,23), the percentage of exchange interactions for the HSE06 functional used here was 25% for the Hartree–Fock mixing. This value represents the mixture of the exact Hartree–Fock exchange with 75% of the PBE functional. Additionally, it is worth specifying the combination of PBE potentials used in the calculations. This can be altered from the PBE0 functional to HSE06 using the HFSCREEN flag in VASP. Here, a value of 0.207 was adopted.

We used the projector augmented wave (PAW) method,^24^ through Vienna ab initio simulation package (VASP)^25,26^ to solve the Kohn–Sham equations (see Section 1 of the Supporting Information file^27^ giving additional computational technical details on the PAW projectors). We optimized the monolayer GaP crystal structure via stress tensor and atomic forces optimization until the atomic forces were less than 0.01 eV/Å with a plane wave cutoff of 510 eV (see Section 2 of the Supporting Information file^27^ giving the optimized structural data). For the other investigated optoelectronic properties, we assumed a cutoff of 286.9 eV. For the Kohn–Sham self-consistent cycle, we adopted a total energy convergence criterion of 10^–6^ eV. For the Brillouin zone integration, we used a **k**-mesh automatic generation scheme through the Monkhorst–Pack method,^28^ with a **k**-points density in the in-plane lattice vector directions of 80 Å^–1^ for the DOS and 40 Å^–1^ for the other properties. To avoid spurious interactions between the monolayer and its periodic images, a vacuum thickness of 20 Å was taken in the unitary cell.

Phonon properties were conducted using the density functional perturbation theory (DFPT) method,^29^ as implemented in the PHONOPY code.^30^ The elastic constants of monolayer GaP were derived in accordance with Hooke’s law utilizing the VASPKIT package.^31^ The calculation involved determining each elastic constant component by analyzing the first-order derivative of the stress–strain curve, which was constructed based on six incremental strains spanning from −1.5 to 1.5% within the material’s elastic regime. Ab initio molecular dynamics simulations were implemented using the canonical ensemble with the temperature regulated by the Andersen thermostat^32^ using a 3 × 3 × 1 supercell. Each molecular dynamics simulation was performed for 10 ps with a time step of 1 fs.

The computational analysis of Raman shift and infrared spectra also involved the application of DFPT,^29^ but now utilizing the PWSCF package within the Quantum Espresso suite.^33^ The pseudopotentials employed in the calculations were generated using the projector augmented wave (PAW) method in the local density approximation (LDA).^24,34^ A kinetic energy cutoff of 75 Ry was employed, along with a **k**-grid consisting of 30 × 30 × 1 **k**-points.

Excitonic and optical properties were performed using WanTiBEXOS code,^35^ that used a maximally localized Wannier function tight-binding (MLWF-TB) Hamiltonian, directly obtained through HSE06 DFT simulation by Wannier90 package,^36^ considering s and p orbital projections for Ga and P chemical elements. Details about the Wannierization formalism and the reliability of the method were exhaustively shown in the last 20 years.^36−41^ The TB Hamiltonian can be obtained using the VASP Wannier90 interface with the input available in Section 4 of the Supporting Information file.^27^ We calculated the linear optical response at two approximations: independent particle approximation (IPA) and the Bethe–Salpeter equation (BSE).^35,42^ The former was obtained at the single particle level, and for the latter, it was considered excitonic effects, where electrons and holes are bonded by Coulomb interaction. BSE was solved using the 2D Coulomb truncated potential (V2DT).^43^ For the absorption spectrum (BSE and IPA), it was taken a **k**-points density of 120 Å^–1^, whereas for the optical activity in the Brillouin zone, it was adopted a **k**-points density of 400 Å^–1^. A smearing of 0.05 eV was assumed to obtain the real and imaginary parts of the dielectric function in both the BSE and IPA approximations. The optical properties at the BSE and IPA levels were calculated considering the lowest three conduction bands and the two highest valence bands. The electron/hole effective mass tensor was obtained by MLWF-TB HSE06 Hamiltonian using finite difference method and a **k**-points distance of 0.001 Å^–1^.

The solar harvesting efficiency was obtained by calculating the power conversion efficiency (PCE) at the Shockley–Queisser (SQ) limit^44^ and with the spectroscopy-limited maximum efficiency (SLME) method,^45^ considering the AM1.5G to model the solar emission spectrum.^46^ At SQ-limit formalism, the recombination fraction fr is not taken in account. Complementary, the calculated properties at the SLME level were done by considering that all incident photons with energy higher than the optical band gap were absorbed, identifying it here as SLME_max_. This approximation motivated the work of Jariwala et al.^47^ in which they showed strategies to enhance the monolayer absorbance rate closer to 100%. These calculations were performed with and without excitonic effects, considering the solar device at 298.15 K. The absorbance was evaluated by considering the material thickness and the total absorption coefficient, which is obtained by the sum of the dielectric function diagonal components. For lamellar materials, such as graphene, due to their 2D nature, the material layer thickness has an atomic size, i.e., very close to 0 Å, which would make the direct use of the standard absorbance expression for bulk systems unfeasible.^45^ To overcome this issue and consequently estimate the absorbance for 2D systems, Bernardi and co-workers^48^ were the first to propose a theoretical framework by assuming the layer thickness of 3.3 Å for the case of graphene, which basically corresponds to the van der Waals length. As reported in refs (49−51), this approach has been successfully employed for different 2D systems. Based on that, in the case of monolayer GaP that has a buckled lattice structure, one needs to add the layer thickness (0.5 Å) to the van der Waals length (3.21 Å), thus resulting in the monolayer GaP thickness of 3.71 Å. More details about the SLME and SQ formalism can be found in refs (35,51).

## 3 Results and Discussion

### 3.1 Structure and Stability

**Figure 1:**
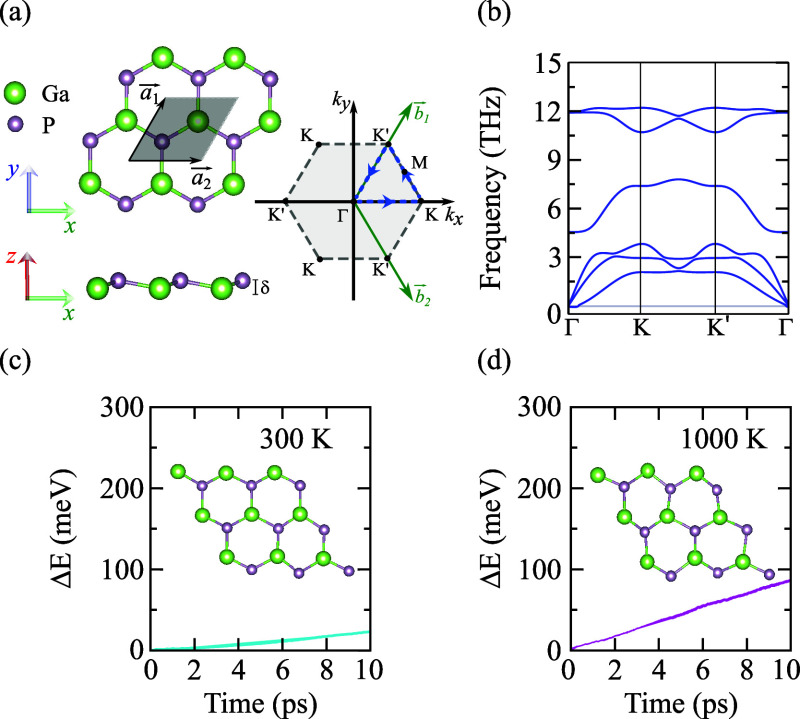
(a) Top and side views of the monolayer GaP crystal structure and the first Brillouin zone. The unit cell is represented by the gray shaded area delimited by the lattice vectors *a⃗*_1_ and *a⃗*_2_. The green and purple spheres represent Ga and P atoms, respectively. In the first Brillouin zone, the high symmetry points, the reciprocal lattice vectors *b⃗*_1_ and *b⃗*_2_, and the path in *k*-space (blue dashed line) used for plotting the bands. (b) Phonon dispersion relation of monolayer GaP along the high-symmetry Γ–*K*–*K*′−Γ path of the hexagonal reciprocal lattice. Ab initio molecular dynamics simulations of monolayer GaP at (c) 300 and (d) 1000 K, exhibiting the variation of the total energy Δ*E* as a function of the time. The images inserted in panels (c) and (d) are snapshots of the crystal structures taken at 10 ps.

We started our investigation by analyzing the structural properties and the stability of pristine monolayer GaP. Figure 1a shows a schematic view of the fully relaxed structure of the single layer GaP. Similar to graphene, it exhibits a hexagonal geometry, here with a space group *P*3*m*1, or equivalently to group no. 156 [see top view in Figure 1a], but now composed by two sublayers displaced by δ = 0.41 Å in the perpendicular direction [see side view in Figure 1a]. In this structure, each Ga atom is covalently bonded to three P atoms and vice versa, with a bond length of 2.29 Å. The lattice constants of the monolayer GaP are *a*_1_ = *a*_2_ = 3.91 Å. The real [*a⃗*_1_ and *a⃗*_2_] and reciprocal [*b⃗*_1_ and *b⃗*_2_] lattice vectors are depicted in Figure 1a, as well as the first Brillouin zone indicating the high-symmetry points and the adopted path in the *k*-space to plot the vibrational, electronic, and excitonic bands shown through this work.

The efficiency of the conjugate gradient optimization algorithm in ensuring the stability of 2D honeycomb structures is not always guaranteed. Hence, it becomes imperative to employ diverse tests to validate the stability in such materials. Thus, in this context, our analysis here was focused on the stability assessment of the 2D GaP honeycomb structure, accomplished through phonon dispersion calculations and ab initio molecular dynamics, as discussed next.

Figure 1b shows the 2D GaP monolayer phonon dispersion relation calculated for the honeycomb structure. Analogous to the phonon dispersion observed in graphene,^52^ the GaP sheet exhibits six phonon branches (three acoustic and 3*N* – 3 optical phonon modes, with *N* being associated with the dimensionality). All calculated phonon modes are positive, indicating stability across the Brillouin zone. Minor deviations, such as slightly negative frequencies, as observed near the Γ-point at low frequencies (see the state below the gray line for null frequency), are attributed to numerical noise. These findings suggest dynamic stability for the 2D GaP monolayer.

Figure 1c,d present the results of ab initio molecular dynamics simulations at 300 and 1000 K, respectively. The plots show the variation of the total energy (Δ*E*) concerning the simulation time, i.e., the difference between the total energy at a specific time and temperature and the total energy at the beginning of the calculation at exactly 300 K. The snapshots in the insets of Figure 1c,d illustrate the last configurations at 10 ps. From Figure 1c, one notices that no structural distortion, bond breaking, or phase transition is observed at 300 K. Even when the structure is heated up to 1000 K, the structure remains roughly intact without pronounced lattice deformation, demonstrating the excellent thermal stability of the hexagonal monolayer GaP (see Section 3 of the Supporting Information file^27^ with additional plots of the variation of the total energy).

### 3.2 Electronic Properties

**Figure 2:**
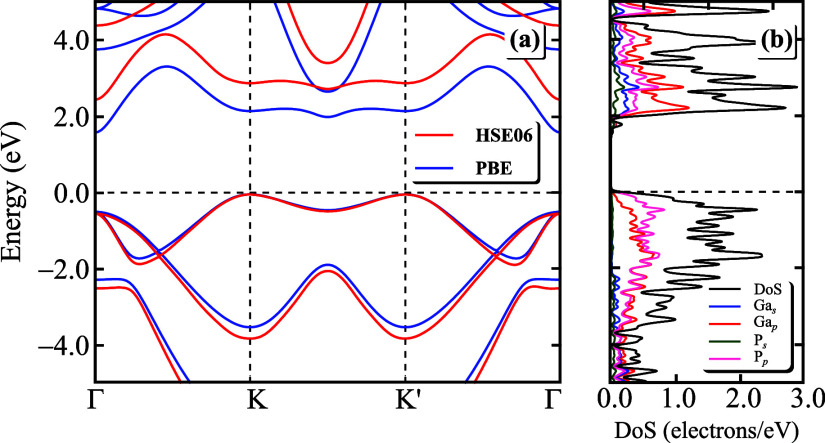
(a) Monolayer GaP band structure as the PBE (blue curves) and HSE06 (red curves) approaches. (b) DOS (black curves) and PDOS at the PBE level. Ga-s and Ga-p orbital projections are shown in blue and red curves, respectively, and P-s and P-p orbital projections in green and violet curves, respectively. The Fermi level is set at 0 eV.

Once we confirm the stability of hexagonal monolayer GaP, we can subsequently safely investigate its electronic properties. As mentioned in Section 2, for that, we employed two functionals: GGA-PBE (blue curve), and HSE06 (red curve). Figure 2 displays (a) the monolayer GaP band structure using both functionals and (b) the DOS (black curve) and the PDOS at the PBE level. The energetic difference in the GaP band gap is a result of employing two distinct functionals, with a band gap value of 1.62 eV when using GGA-PBE and 2.49 eV with HSE06. However, by using both functionals, one observes that the hexagonal monolayer GaP gap is indirect with the highest value on the valence band at the *K*/*K*′-point and the lowest energy value on the conduction band at the Γ-point. Note that apart from a band shifting, the band structures obtained using both functionals exhibit equivalent physical features, such as band curvatures, the double degeneracy for the lowest hole states at the Γ-point, and band crossing along the *K*–Γ/*K*′−Γ path. Notably, this characteristic of an indirect band gap semiconductor has been reported in ref (16), where they inferred an indirect gap of 1.97 eV at *K*–Γ and a direct gap of 2.28 eV at the *K* point, closely resembling reported direct band gap values for zinc blende and buckled structures of GaP. Furthermore, by examining the different orbital projected contributions at the PDOS in Figure 2b, one can realize that the Ga-p and P-p orbitals exhibit a greater contribution than the *s* orbitals, both in the valence band maximum and the conduction band minimum.

Analyzing the band structure curvature and computing the effective mass tensor elements , where {*i*, *j*} denotes the reciprocal components and the subscript *n* being the band index, of the lowest (highest) energetically conduction (valence) band in Figure 2a around Γ(*K*)-point, one can obtain electron and hole effective masses close to the Fermi energy that, in turn, are relevant for developing subsequent effective models for charge carriers in monolayer GaP. Here, such derivative quantities were evaluated numerically using the finite difference method around the Γ and *K* points for obtaining the electron and hole masses, respectively, following the corresponding paths near the mentioned high-symmetry points along *k*_*x*_, *k*_*y*_, and diagonal directions to get the masses *m*_*xx*_, *m*_*yy*_, and *m*_*xy*_, respectively [see the first Brillouin zone in Figure 1a]. Such computed effective mass tensor elements are depicted in Table 1 for both electron and hole *m*_*xx*_, *m*_*yy*_, and *m*_*xy*_ elements. By comparing the hole values presented in Table 1 for the *xx* and *yy* directions, one can clearly see an indication that the monolayer GaP has an anisotropic band close to the valence band maximum, since it exhibits a direction-dependent hole effective mass. By analyzing the electron effective masses along the calculated directions, we at first are led to think that the conduction band minimum is isotropic around the Γ-point since *m*_*xx*_^e^ = *m*_*yy*_^e^. However, the diagonal tensor element is different, i.e., *m*_*xy*_^e^ ≠ *m*_*xx*_^e^ and *m*_*xy*_^e^ ≠ *m*_*yy*_^e^. Therefore, both conduction and valence monolayer GaP bands are anisotropic with high effective mass magnitude at the *xy*-direction. As a consequence of the band structure anisotropy, many physical observables of monolayer GaP would also exhibit anisotropic/asymmetric behavior, similar to what is observed in the transport,^53^ optoelectronic,^54−59^ and excitonic^60,61^ properties of multilayer black phosphorus; thus being an important exploratory direction for future studies in monolayer GaP.

**Table 1:** Electron and Hole 2D Effective Mass Tensor Elements *m*_*ij*_ with *i*, *j* ≡{*x*, and *y*} in Units of the Bare Electron Mass *m*_0_

	electron (*m*_0_)	hole (*m*_0_)
*m*_*xx*_	0.13	0.58
*m*_*yy*_	0.13	0.80
*m*_*xy*_	9.33	33.87

### 3.3 Elastic Properties

The elastic properties of monolayer GaP are characterized by four independent elastic constants: *C*_11_ = 31.741 GPa, *C*_22_ = 31.951 GPa, *C*_12_ = 11.186 GPa and *C*_66_ = 10.286 GPa. To validate the mechanical stability of monolayer GaP, these constants *C*_*i*_ (with *i* = [1, 6]) should satisfy the Born criteria for 2D lattices^62,63^ given by
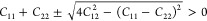
1

Additionally, *C*_66_ > 0 further supports the mechanical stability. Furthermore, the determined elastic constants can be utilized to estimate the orientation-dependent Young’s modulus (*E*), shear modulus (*G*), and Poisson’s ratio (ν) of monolayer GaP using the following equations:
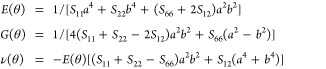
2where *a* = cos(θ) and *b* = sin(θ), with θ representing the orientation angle defined with respect to the *x*-axis. The compliance constants *S*_11_, *S*_22_, *S*_12_, and *S*_66_ in eq 2 are determined by inverting the elastic constants matrix, i.e., *C* = *S*^–1^.

**Figure 3:**
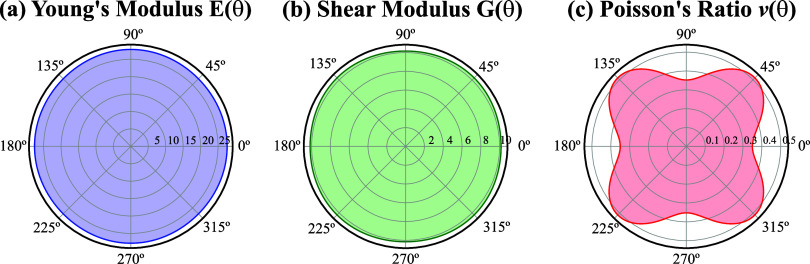
Orientation dependence of (a) the Young’s modulus *E*(θ), (b) the shear modulus *G*(θ), and (c) the Poisson’s ratio ν(θ) for a monolayer GaP obtained via DFT calculations.

Graphical representations in the polar coordinate θ of the elastic parameters *E*(θ), *G*(θ), and ν(θ) are illustrated in Figure 3. All three investigated mechanical quantities, except for Poisson’s ratio, demonstrate isotropic behavior, as noticed by a circularly symmetric plot in the θ-coordinate. Young’s modulus is quantified at 27.879 GPa, while the shear modulus is determined to be 10.320 GPa. Notably, Young’s modulus of monolayer GaP is significantly lower than that of most other 2D carbon allotropes, such as the approximately 1 TPa of graphene^64^ and the approximately 600 GPa of graphyne.^65^ According to this comparison, this suggests that monolayer GaP is more flexible than 2D carbon-based materials.

A nonmonotonic variation is discerned in Poisson’s ratio of monolayer GaP as θ varies from 0° to 90°, as shown in Figure 3c. Specifically, ν increases from its minimum value of 0.35 to its maximum value of 0.51 as θ changes from 0° to 45°. The Poisson’s ratio of monolayer GaP is comparatively smaller than that of other existing 2D carbon-based materials, such as graphene (ν = 0.3)^66^ and graphyne (ν = 0.42).^67^ The nature of the anisotropic Poisson’s ratio can roughly be understood by the crystallographic aspect of the monolayer GaP. Note from Figure 1a that monolayer GaP can be viewed as two sublayers displaced by a non-null vertical distance δ, with one of them being formed by Ga atoms and the other by P atoms. Due to this vertical displacement of the two sublayers, lattice distortions will affect the 2D GaP crystal differently depending on the strain/stress applied direction, i.e. one can see this statement as a direction-dependent resistance to deform, depending on the number of out-of-plane hoppings that will be affected by the applied deformation. A very interesting example of a 2D material with electronic and mechanical anisotropy that also exhibits, similar to what we obtained here for monolayer GaP, orientation-dependent Poisson’s ratio^68,69^ is the monolayer black phosphorus. According to ref (68), the Poisson’s ratio along zigzag and armchair directions are 0.703 and 0.175, respectively, showing its strong direction-dependent mechanical feature.

### 3.4 Raman and Infrared Spectra

**Figure 4:**
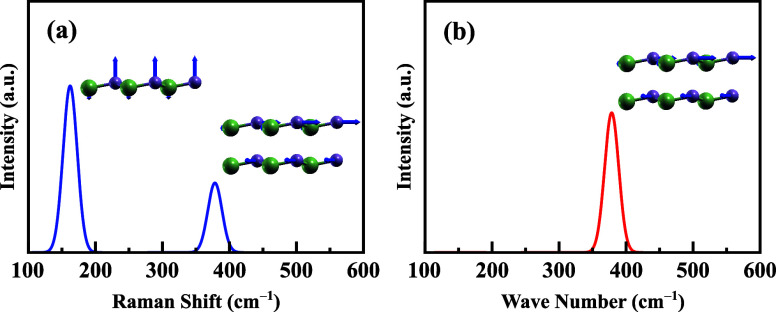
(a) Monolayer GaP Raman spectrum, exhibiting two pronounced peaks at frequencies ≈150 and ≈382 cm^–1^ associated with the out-of-plane and in-plane vibrations’ modes, respectively. (b) Monolayer GaP infrared spectrum with a double degenerate peak at ≈382 cm^–1^. Insets illustrate the corresponding lattice structures’ vibration direction, denoted by the blue arrows.

To characterize the chemical, vibrational, and optical bonding states of hexagonal monolayer GaP, we conducted theoretical studies using Raman and infrared measurements. The results are presented in Figure 4a,b. Figure 4a highlights three active optical modes in the Raman spectrum. Two of these vibrational modes are degenerate, originating from in-plane vibration with a frequency of ≈380.2 cm^–1^. The right insets of Figure 4a illustrate the two in-plane vibration directions, as indicated by the blue arrows. Additionally, one observes a resonant peak with lower intensity at a frequency of ≈150 cm^–1^ associated with the out-of-plane vibration mode, as illustrated in the left inset of Figure 4a. To complement our analysis, we examined the infrared spectrum, as shown in Figure 4b. The infrared spectrum displays a single peak with two degenerate peaks at 380.2 cm^–1^, representing in-plane vibration modes, similar to the in-plane Raman modes observed in Figure 4a. Contrary to the Raman case, the out-of-plane mode is found to be inactive in the infrared spectrum.

The experimental study reported in ref (70), conducting micro-Raman analysis and FTIR reflectance determination on porous layers, based on *n* type crystals grown on (111) GaP and (100) InP LEC, carried out more than two decades ago, showed indications of LO and TO phonon modes in the GaP crystal at 402.3 and 366 cm^–1^. The reported resonant frequencies for bulk cases in ref (70) are higher than the ones calculated here for monolayer GaP, showing a clear dependence on the number of layers in such physical properties as also observed in lamellar van der Waals systems^71,72^ (see Section 5 of the Supporting Information file^27^ showing the bulk GaP Raman spectrum obtained via our methodological approach and additional discussions comparing it with the one reported in ref (70)). Furthermore, the measured spectra in ref (70) exhibited an additional F mode positioned between the TO and LO phonon frequencies of bulk GaP, attributed to the presence of pores. To the best of our knowledge, no Raman and infrared theoretical calculations for monolayer GaP have been reported in the literature so far.

### 3.5 Excitonic and Optical Properties

**Figure 5:**
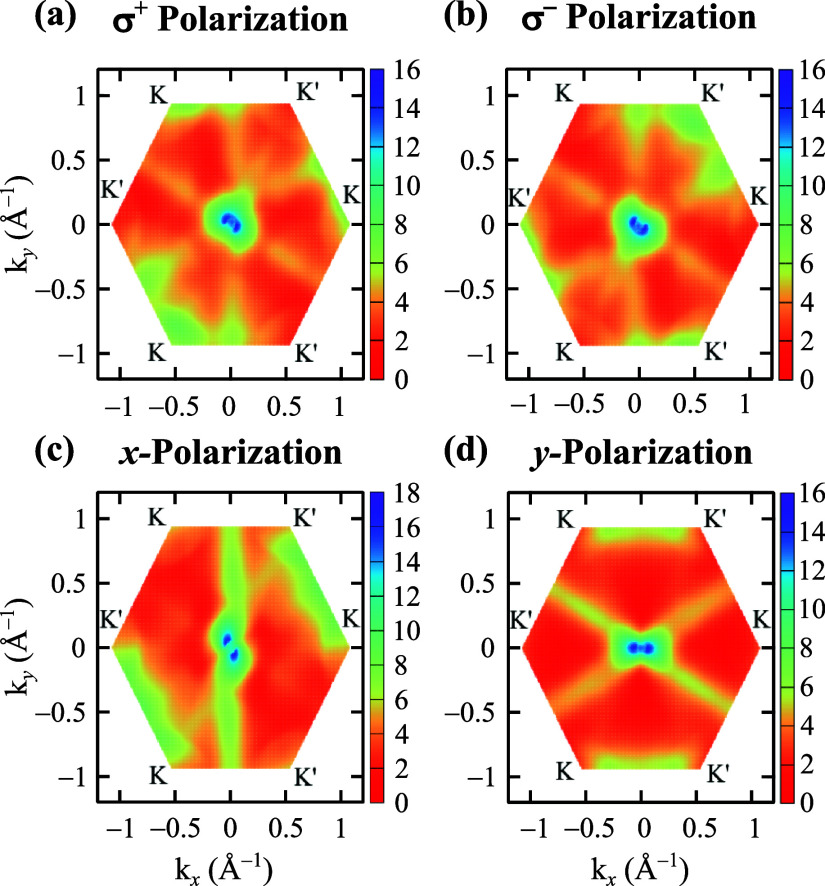
Monolayer GaP optical activity (Å^2^) with (a, b) circular and (c, d) linear light polarization shown in the first Brillouin zone. All calculations were performed with DFT-HSE06 and MLWF-TB parametrization.

The single-particle optical activities in the first Brillouin zone are shown in Figure 5a, b for circularly polarized light and in Figure 5c, d for linearly polarized light (see Sections 6 and 7 of the Supporting Information file^27^ showing additional plots of the monolayer GaP Berry curvature and the circular and linear optical dichroism). These properties were calculated in the IPA scope by summing the oscillator strength for all possible direct optical transitions, considering the three lowest conduction bands and the two highest valence bands. Generally speaking, by comparing σ^+^ [Figure 5a] and σ^–^ [Figure 5b] results for circular light polarization, as well as [Figure 5c] *x*- and [Figure 5d] *y*-results for linear light polarization, one can observe that monolayer GaP exhibits direction-dependent selection rules for both circular and linear light polarization cases. Moreover, it is evident from Figure 5 that regardless of the light polarization direction, the optical activities are higher in the vicinity of the Γ-point and exhibit an anisotropic behavior with an asymmetric peanut-like shape (see the blue region in the contour plot of Figure 5). In addition to the high intensities around the Γ-point, some partial *K*–*K*′ valley selections can be observed for circular light polarization, with non-null optical activities for both polarization directions presenting a higher intensity around the *K* (*K*′) point for the σ^+^ (σ^–^) case. This *K*–*K*′ valley selection rule has a similar behavior as shown for hexagonal monolayer TiBr_2_^73^ and group VI 2H transition metal dichalcogenides.^74^ For these mentioned systems, different from monolayer GaP, one has a total valley selection rule under optical helicity.

**Figure 6:**
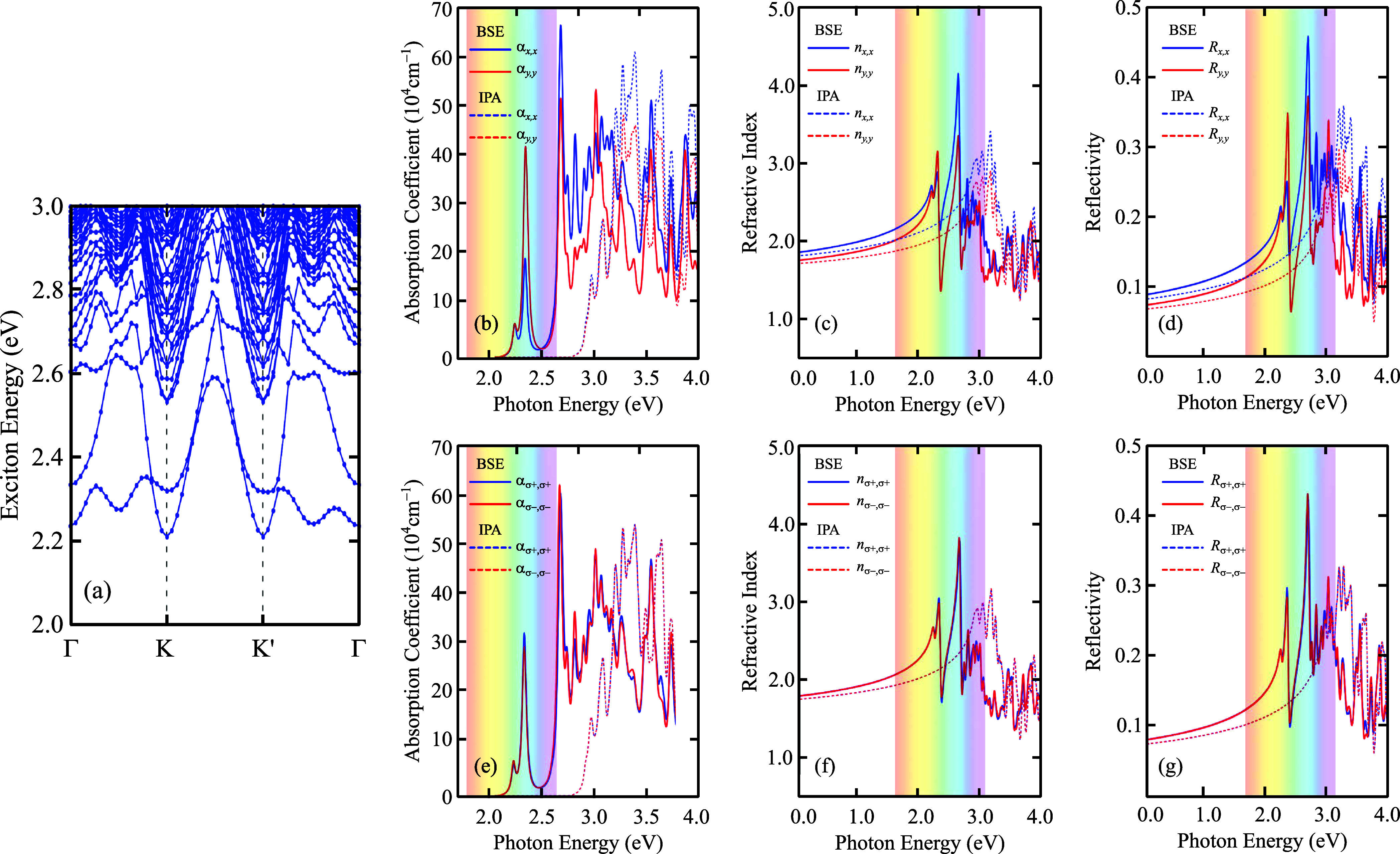
(a) Monolayer GaP exciton band structure by using DFT-HSE06 and MLWF-TB+BSE. Monolayer GaP optical properties: (b, e) absorption coefficient, (c, f) refractive index, and (d, g) reflectivity. These quantities were calculated within BSE (solid curves) and IPA (dashed curves) approximations for [top panels in (b–d)] linear and [bottom panels in (e–g)] circular light polarizations. Blue and red curves correspond to *x̂* (σ^+^) and *ŷ* (σ^–^) linearly (circularly) polarized light cases, respectively.

Beyond the single-particle picture, it is interesting to explore excitonic effects. For that, we calculated the exciton band structure within the BSE approximation, as mentioned in Section 2. The resulting excitonic bands for the monolayer GaP are shown in Figure 6a. Concerning the understanding of excitonic band structures, it is worth emphasizing that excitonic states are not characterized into “conduction” and “valence” excitonic states, as electron–hole charge particle states are usually featured for the case of the electronic band structures since exciton energy is obtained by the difference between electron and hole energies minus the binding energy from Coulomb-like interaction. If the Coulomb interaction were set to zero, the resulting exciton band structure would be equivalent to mapping the direct band gap in the Γ point and all indirect band gaps in the rest of the **k** path. This kind of result is not very common in the literature, as the computational costs to obtain the exciton band structure using first-principle methods are very expensive. For example, the previous refs (35,74,75) provided excitonic band structures for different systems using BSE+TB methods. One can observe from Figure 6a that the excitonic ground state is indirect and double degenerate at the *K* or *K*′ exciton momenta with an energy of 2.21 eV. This feature can be understood from the indirect band gap of the single-particle electronic band structure. Analyzing a possible direct transition, one notices that the excitonic direct ground state happens for an exciton momentum at the Γ-point with an energy of 2.24 eV. From the excitonic bands [Figure 6] and the single-particle bands [Figure 2a], we can estimate the exciton binding energy by computing the difference between the fundamental electronic band gap and the exciton ground state. It results in a value of 275 meV, presenting a magnitude in a similar energetic range as the one obtained for other semiconductor 2D materials; For instance, transition metal dichalcogenides and black phosphorus have binding energies on the order of hundreds of meV, which, in turn, allows their exciton state measurements at room temperature through photoluminescence and absorption spectroscopy.^60,74,76−80^

The absorption spectrum, refractive index, and reflectivity at BSE (solid curves) and IPA (dashed curves) calculation levels with linear and circular light polarizations are shown in Figure 6b–d and e–g, respectively. By analyzing the circularly polarized light case, one notices that all three calculated optical properties are isotropic regardless of the level of calculation (BSE or IPA), i.e., α_σ^+^σ^+^_ = α_σ^–^σ^–^_, *n*_σ^+^σ^+^_ = *n*_σ^–^σ^–^_, and *R*_σ^+^σ^+^_ = *R*_σ^–^σ^–^_. At first glance, this isotropy for the circularly polarized light results (bottom panels in Figure 6) might seem in disagreement with the optical activity results shown in Figure 5a,b, since the optical activities in Figure 5a,b present a nonequivalent optical response between *K* and *K*′ for a fixed σ^±^–polarization. However, by comparing Figure 5a,b for σ^+^ and σ^–^, one can realize that these optical activities exhibit a π-rotated (*C*_2_) symmetry, such that it obeys the following equivalence OA(σ^+^, *K*) = OA(σ^–^, *K*′) between *K* and *K*′ results for different polarizations, with OA denoting the optical activity. In addition, note from Figures 2a and 6a that the electronic and excitonic levels have the same energies at both the *K* and *K*′ valleys. Thus, the interplay between the mentioned *K* – *K*′ relation and their similar energies results in the same optical response for σ^+^ and σ^–^, as shown in the absorption spectra in Figure 6e.

On the other hand, the linear optical responses shown in Figure 6b–d present an anisotropic behavior, such that α_*xx*_ ≠ α_*yy*_, *n*_*xx*_ ≠ *n*_*yy*_, and *R*_*xx*_ ≠ *R*_*yy*_. Moreover, note that neither circular nor linear light polarizations change the optical band gap, which is 2.92 eV at the IPA and 2.24 eV at the BSE levels. It is worth mentioning that the difference between the band gap energy obtained at IPA and BSE levels is related to the strong exciton effects accounted for via the electron–hole Coulomb-like interaction, which causes an energetic red-shift at the BSE level, diminishing the optical band gap. The refractive index is very sensitive to linear light polarization [Figure 6b], being easy to differentiate it for *x̂* (blue curves) and *ŷ* (red curves) light polarizations. Its value for the linearly polarized light case is slightly lower in the *ŷ* direction with a maximum peak closer to 2.5 eV at BSE and 3.5 eV at IPA. Note from Figure 6c that the reflectivity for light excitation energies higher than or equal to the optical band gap exhibits higher magnitudes, with a maximum value closer to 45% at BSE and 30% at IPA. It shows that quasi-particle effects enhance the optical reflectivity of monolayer GaP. These optical reflectivity values suggest applications for monolayer GaP regarding light reflection, which could also be tuned depending on the light polarization.

### 3.6 Insights on Solar Harvesting Efficiency

Monolayer GaP solar harvesting efficiency was obtained through the calculation of PCE, which is straightforwardly computed from the optical band gap using the SQ Limit^44^ and the SLME method^45^ that, in turn, depends on the fundamental band gap, the optical band gap, and the absorption spectrum. The PCE at both approximations was obtained with and without excitonic effects. When quasi-particle effects are taken into account, the optical band gap is estimated from the direct exciton ground state, whereas the fundamental band gap is estimated from the exciton ground state obtained from the exciton band structure. It is also important to remember that the efficiencies estimated by these methods are the upper limit of the solar harvesting efficiency of monolayer GaP, which would require a sophisticated experimental investigation to achieve such values. The applied methods to estimate the PCE in this work do not consider the role of exciton binding energy in solar energy conversion; however, this can prevent electron–hole pair separation and transport for the electron transport layer and hole transport layer in the solar cell architecture. Therefore, it is expected that this feature could reduce the solar harvesting efficiency of 2D materials.

From the SLME approach, the PCE obtained with IPA is 1.43%. When excitonic effects that reduce the optical band gap are considered, the PCE goes to 3.94%; this small value for efficiency is justified due to the small thickness of the sample that is composed of a monolayer crystal. As a consequence, it results in a lower light absorbance and a small fraction of the incident light being absorbed by the material. Jariwala and co-workers showed in ref (47) that applying light trapping techniques can enhance light absorbance rate to values close to 100%, leading to a massive boost in the solar harvesting performance. Considering this scenario, we also estimated the PCE at the SLME_max_ approximation. The absorbance curve was changed to a Heaviside function, which is null for photons with energy lower than the optical band gap and 1 otherwise, being exactly done in the SQ-limit. However, at SLME_max_, the recombination fraction fr is also considered, which results in values smaller than the SQ-limit. As the values of fr are smaller, the PCE values from SLME_max_ and the SQ-limit are very close. At the IPA level, we obtained a solar harvesting efficiency of 4.50 and 17.30% at the BSE level, which provides a more realistic description of the optical response (see Section 8 of the Supporting Information file^27^ presenting additional data concerning the solar harvesting efficiency). According to the obtained results here, one can notice that monolayer GaP shows a solar harvesting efficiency comparable with group VI transition metal dichalcogenides, a class of materials that has been exhaustively investigated for photovoltaic devices.^74,81,82^

## 4 Summary and Final Remarks

In summary, we have theoretically investigated the electronic, mechanical, and optical properties of monolayer GaP using first-principles calculations and ab initio molecular dynamics. The reported physical properties here were computed by taking excitonic effects into account and comparing them to the situation in the absence of them. In addition, general insights on solar harvesting efficiency were also reported since monolayer GaP is a semiconductor and can be used as a promising component of photovoltaic devices.

From the structural properties, our system stability was assured from the lack of imaginary frequencies at phonon dispersion, from the lack of broken bonds and lower total energy variation in ab initio molecular dynamics, and also from the elastic constant’s stability conditions. Mechanically, GaP exhibited significant strength, as evidenced by its impressive tensile strength, Young’s modulus, and hardness. These mechanical properties position GaP as a suitable candidate for applications requiring robust and resilient materials. Electronically, GaP showcases exceptional characteristics, including favorable electrical conductivity and an ideal band gap for semiconductor devices. Such attributes make GaP highly promising for high-performance electronic applications.

From the vibrational properties, three optical vibrational modes were viewed to be Raman active with a double degenerate in-plane mode, and in the infrared spectrum, only the double degenerate mode was shown to be active. From the optical properties, our system showed a partial valley selection rule under optical helicity; however, due to the valley symmetry, the optical response under circular light polarization was shown to be independent of the σ_±_ choice. When linear light was considered, the optical responses were slightly different, presenting the same transition peaks but with different absorption intensities when excitonic effects were taken into account. These quasi-particle effects showed a significant red-shift in the optical band gap, with GaP exhibiting an exciton binding energy of 275 meV. Owing to this optical band gap in the visible region, we showed that the monolayer GaP has a good potential for solar harvesting, resulting in a power conversion efficiency of 17.30% when light trapping techniques were applied, making this material interesting for flexible solar cells. These optical features highlight GaP’s potential for use in optoelectronic devices, including LEDs, photodetectors, and solar devices.

Overall, our findings mark a significant advancement in the understanding of GaP’s properties and highlight its versatility for a wide range of electronic and optical applications. Further exploration of the practical applications of these discoveries will undoubtedly contribute to the development of innovative technologies in various fields.

## Supporting Information Available

Description of (1) PAW projectors: computational technical details, (2) optimized structural data (POSCAR), (3) ab initio molecular dynamics, (4) INCAR input for Wannierization, (5) bulk GaP Raman spectrum, (6) Berry curvature, (7) excitonic and optical properties: optical activity in BZ, and (8) insights into solar harvesting efficiency; additional results and data in Figures S1–S4 and Tables S1–S2 (PDF)
