# Supplementary material for: Stability and Optoelectronic Properties of Two-Dimensional Gallium Phosphide
Source: ACS Omega. 2024 Aug 9;9(33):35666–75. doi: 10.1021/acsomega.4c03861 (PMC11339990; doi:10.1021/acsomega.4c03861)
Supplement: Supplementary file 1 — ao4c03861_si_001.pdf [file ao4c03861_si_001.pdf]

Supporting Information: Stability and optoelectronic properties of two-dimensional gallium phosphide

Elisangela da Silva Barboza,[†] Kessia L. M. Cruz,[†] Ramon S. Ferreira,[†,‡,¶] Alexandre C. Dias,[§] Erika N. Lima,[†] Diego R. da Costa,[*,‖,⊥] and Teldo A. S. Pereira[†,¶]

†*Instituto de Física, Universidade Federal de Mato Grosso, 78060-900, Cuiabá, MT, Brazil*

‡*Departamento de Física, Universidade Federal do Piauí, 64049-550, Teresina, PI, Brazil*

¶*National Institute of Science and Technology on Materials Informatics, Campinas, Brazil*

§*Institute of Physics and International Center of Physics, University of Brasília, Brasília 70919-970, DF, Brazil*

‖*Departamento de Física, Universidade Federal do Ceará, Campus do Pici, 60455-900 Fortaleza, Ceará, Brazil*

⊥*Department of Physics, University of Antwerp, Groenenborgerlaan 171, B-2020 Antwerp, Belgium*

E-mail: diego_rabelo@fisica.ufc.br

Contents

1 **PAW Projectors: Computational Technical Details** S-2

2 **Optimized Structural Data (POSCAR)** S-2

3 ***Ab Initio* Molecular Dynamics** S-3

4 **INCAR input for Wannierization** S-3

5 **Bulk GaP Raman spectrum** S-5

6 **Berry Curvature** S-6

7 **Excitonic and Optical Properties: Optical Activity in BZ** S-7

8 **Insights in Solar Harvesting Efficiency** S-7

References S-8

1 PAW Projectors: Computational Technical Details

Table S1: Relevant information about the selected PAW projectors, which includes PAW-PBE projector name, date of projector creation, number of valence electrons, Zval, electron configuration of valence states and maximum recommended cutoff energy, and ENMAX for all selected chemical species.

Element	`POTCAR` PAW-PBE	Date `POTCAR`	Z_{val}	Valence	`ENMAX` (eV)
Ga	Ga_GW	03/22/2012	3	$4s^2$ $4p^1$	134.678
P	P_GW	03/19/2012	5	$3s^2$ $3p^3$	255.040

2 Optimized Structural Data (POSCAR)

```
GaP monolayer

1.000

3.9133046481234364 0.0000000000000000 0.0000000000000000
```

```
-1.9566523240116771 3.3899002150620343 0.0000000000000000
0.0000000000000000 0.0000000000000000 20.0000000000000000
Ga P
1 1
Direct
0.3463858143053500 0.6797401668515519 0.5103694339431826 Ga1
0.6796927676946467 0.3463467591484459 0.4896305660568174 P1
```

3 *Ab Initio* Molecular Dynamics

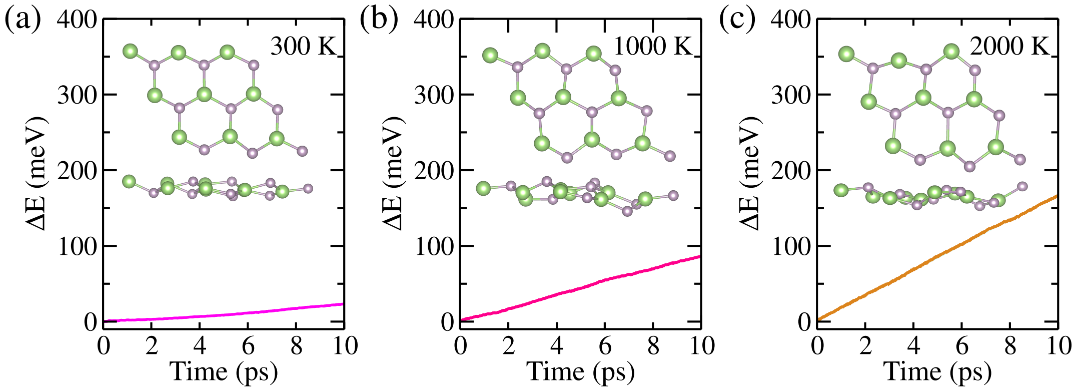

Figure S1: *Ab initio* molecular dynamics simulations of monolayer GaP at (a) 300 K, (b) 1000 K, and (c) 2000 K, exhibiting the variation of the total energy ΔE as a function of the time. The images inserted are snapshots of the crystal structures taken at 10 ps.

4 INCAR input for Wannierization

```
Electronic relaxation
ENCUT = 286.92
ALGO = All
NELM = 200
NELMIN = 6
NELMDL = -12
EDIFF = 1.0E-6
AMIX = 0.1
```

```
BMIX = 0.0001

NBANDS = 16

Calculation mode :

PREC = Accurate

IVDW = 0

ISPIN = 1

ADDGRID = .TRUE.

LASPH = .TRUE.

LHFCALC = .TRUE.

AEXX = 0.25

HFSCREEN = 0.207

Integration over the Brillouin zone (BZ):

ISMEAR = 0

SIGMA = 0.01

DOS calculation:

LORBIT = 10

NEDOS = 6001

OUTCAR size:

NWRITE = 1

LWAVE = .FALSE.

LCHARG = .FALSE.

Key for parallel mode calculation:

KPAR = 4

LPLANE = .TRUE.

LWANNIER90 = .TRUE.

NUM_WANN = 16

WANNIER90_WIN="

Begin Projections

random
```

```
Ga:  s,p

P: s,p

End Projections

use_ws_distance=.false.

num_iter = 2000

kmesh_tol = 0.000001

search_shells = 500

guiding_centres = true

write_hr = T

bands_plot = T

begin kpoint_path

G 0.0 0.0 0.0 K 0.666666 -0.3333333 0.0

K 0.666666 -0.3333333 0.0 K' 0.3333333 0.3333333 0.0

K' 0.3333333 0.3333333 0.0 G 0.0 0.0 0.0

end kpoint_path

"
```

5 Bulk GaP Raman spectrum

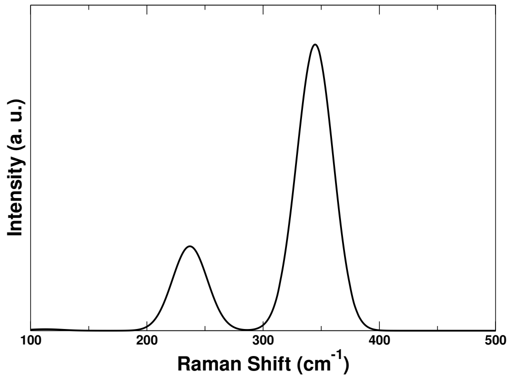

Figure S2: Bulk GaP Raman spectrum, exhibiting two pronounced peaks at frequencies $\approx$ 250 cm^{-1} and $\approx$ 350 cm^{-1} associated with the out-of-plane and in-plane vibrations' modes, respectively.

To show the robustness of our methodological approach and a comparison with the obtained results in Ref.[S1] for bulk GaP case, we show in Fig. S2 the Raman scattering calculations on the zincblende structure of GaP (bulk). In Ref.[S1], the authors performed Raman spectroscopy measurements on a GaP membrane with (111) orientation, observing two characteristic peaks: a TO mode (366 cm^{-1}) and an LO mode (402.3 cm^{-1}). A discussion comparing our obtained results for monolayer GaP and these values obtained by Ref.[S1] for the bulk case was depicted in the second paragraph of Sec. IIID of the main text. In addition, our result in Fig. S2 exhibits good agreement with the experimental findings, as can be noted by Refs.[S2–S5], both in terms of frequency range and mode intensity. By comparing the obtained resonance values in Fig. S2, one notes that the LO mode shows greater intensity than the TO mode. Conversely, in Ref.[S1], the TO mode demonstrates higher intensity relative to the LO mode. This discrepancy between Fig. S2 and Ref.[S1] arises because Raman scattering is influenced by several factors, including the polarization and direction of the incident light, the crystal symmetry and orientation of the sample, and the direction and polarization of the scattered light[S6].

6 Berry Curvature

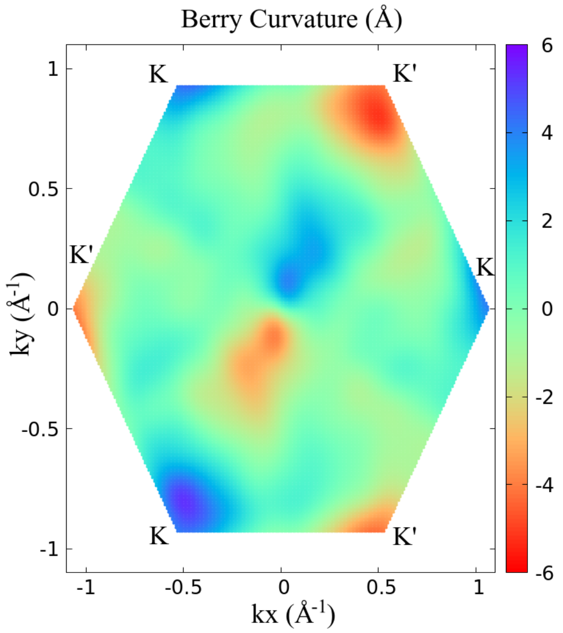

Figure S3: Monolayer GaP total Berry curvature (DFT-HSE06 MLWF-TB parametrization) obtained in the first Brillouin zone.

7 Excitonic and Optical Properties: Optical Activity in BZ

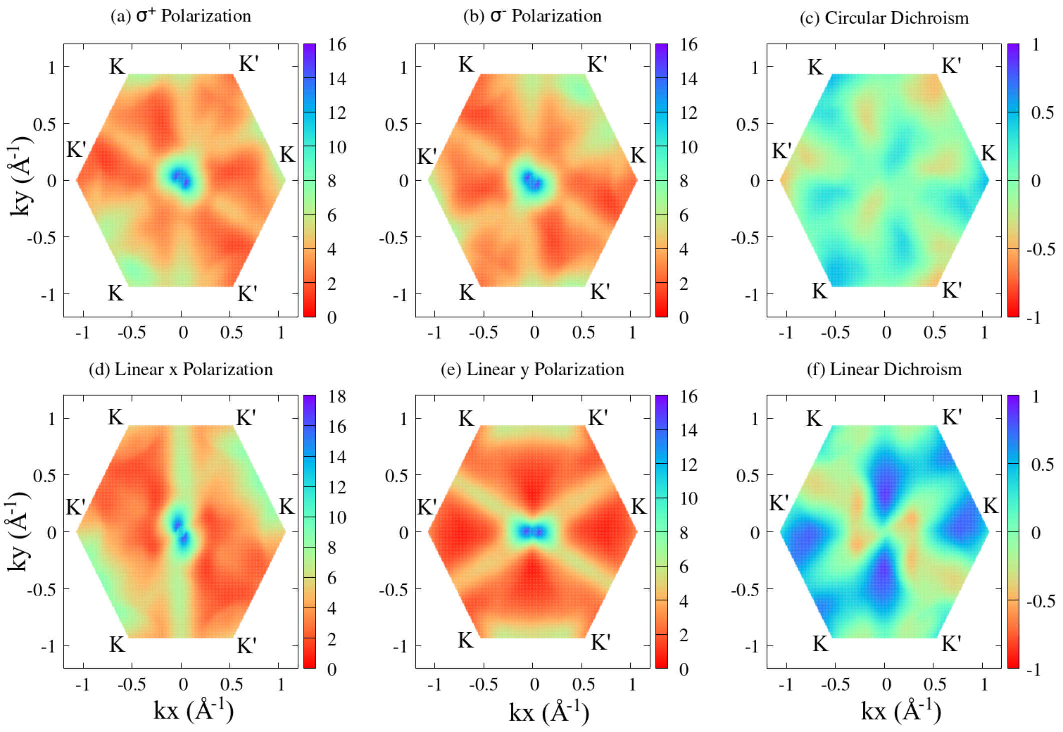

Figure S4: Monolayer GaP optical activity shown in the first Brillouin zone, with (a)-(b) circular light polarization and with (d)-(e) linear light polarization. (c) Circular and (f) linear optical dichroism are shown in the first Brillouin zone. All calculations were performed with DFT-HSE06 and MLWF-TB parametrization.

8 Insights in Solar Harvesting Efficiency

Table S2: Maximum achieved PCE at IPA and BSE level, short circuit current density, J_{sc} (W/Vm^2), open circuit voltage, V_{oc} (V), fr recombination factor, FF fill factor (%), power conversion efficiency PCE (%). All properties are obtained at Shockley–Queisser limit (SQ), Spectroscopy Limited Maximum Efficiency (SLME) and SLME considering that 100 % of photon absorbance starts from direct band gap at IPA and exciton bright ground state at BSE levels, calculated with $T = 298.15$ K and considering the monolayer thickness plus vdW length (3.21 Å), which results in 3.71 Å.

	$SLME^{IPA}$	$SLME^{IPA}_{max}$	SQ^{IPA}	$SLME^{BSE}$	$SLME^{BSE}_{max}$	SQ^{BSE}
J_{sc} (W/Vm^2)	7.21	23.06	23.06	22.39	99.90	99.90
V_{oc} (V)	2.12	2.09	2.53	1.89	1.76	1.89
fr	0.00	0.00	-	0.37	0.37	-
FF (%)	93.51	93.44	94.37	92.89	92.80	92.89
PCE (%)	1.43	4.51	5.50	3.94	17.30	17.55

References

(S1) Sarua, A.; Gartner, G.; Irmer, G.; Monecke, J.; Tiginyanu, I.; Hartnagel, H. Raman and IR-Reflectance Spectra of Porous III-V Semiconductor Structures. *Physica Status Solidi (a)* **2000**, *182*, 207.

(S2) Dubey, R.; Dubey, S.; Yadav, A.; Sulania, I.; Kanjilal, D. Raman scattering and FTIR studies of 100 MeV Fe9+ ion-irradiated gallium phosphide. *Radiation Effects and Defects in Solids* **2011**, *166*, 743–748.

(S3) Yashinski, M.; Gutiérrez, H.; Muhlstein, C. On the origins of anomalous elastic moduli and failure strains of GaP nanowires. *Nanotechnology* **2017**, *28*, 065703.

(S4) Ushakov, V.; Dravin, V.; Mel'nik, N.; Zavaritskaya, T.; Loiko, N.; Karavanskii, V.; Konstantinova, E.; Timoshenko, V. Y. Ion implantation of porous gallium phosphide. *Semiconductors* **1998**, *32*, 886–890.

(S5) Omar, K. M.; Khan, Z. H.; Soni, R. K.; Abbi, S. C. The Effect of Etchant Concentration on Surface Morphology Of Porous GaP Produced By Laser-Induced Etching. *Modern Applied Science* **2008**, *2*, 1.

(S6) Krylov, A. Raman Spectroscopy of Crystals. 2020.
